# Association of HLA-DR2-Related Haplotype (HLA-DRB5*01-DRB1*1501-DQB1*0602) in Patients with Multiple Sclerosis in Khuzestan Province

**DOI:** 10.22037/ijcn.v14i4.18795

**Published:** 2021

**Authors:** Nooshin DELFAN, Hamid GALEHDARI, Farideh GHANBARI MARDASI, Rezvan ZABIHI, Tahereh LATIFI PAKDEHI, Tahereh SEIFI, Nastaran MAJDINASAB

**Affiliations:** 1 Department of Genetics, Faculty of Sciences, Shahid Chamran University of Ahvaz, Ahvaz Iran.; 2 Department of Neurology, Jondishapour University of Medical Sciences, Musculoskeletal Rehabilitation Research Center, Ahvaz, Iran.

**Keywords:** Multiple sclerosis, HLA-DRB1*1501, HLA-DR2, PCR-SSP, Iran

## Abstract

**Objective:**

Multiple sclerosis (MS) is a partially heritable autoimmune disease. *HLA-DR2* is the largest identified genetic risk factor for MS. The largest identified genetic risk factor is haplotype from the MHC class II HLA-DR2, which increases the disease risk. The *HLA-DR2* distribution in MS patients has been confirmed, but contradictory outcomes have been found. Moreover, the *HLA-DR2* effect on ethnicity and gender is unclear. There are no data regarding the HLA-DR2 (HLA-DRB1*1501-DRB5*01-DQB1*0602) association with MS in Khuzestan Province, Iran. This study aimed to investigate the association of *HLA-DR2* with MS regarding both sex and ethnicity in this province.

**Materials & Methods:**

A total of 399 individuals were recruited. HLA typing was conducted using the polymerase chain reaction amplification with sequence-specific primers technology. The *HLA-DR2 *association with MS was analyzed, and also its probable association with gender, ethnicity, the expanded disability status scale (EDSS), and MS clinical course was examined using the Chi-square test.

**Results:**

HLA-DRB5*01 ^-^ -DQB1*0602 ^-^ as the most common *HLA* haplotype was found in both patient and control groups. In contrast, the DRB5*01^+^-DRB1*1501^+^-DQB1*0602^-^ frequency was very low in the groups. It was observed that haplotypes had no association with MS susceptibility. Most of the haplotypes showed no association with ethnicity, sex, EDSS, and MS course except for the HLA-DRB5*01^+^-DRB1*1501^+^-DQB1*0602^-^ haplotype that was positively associated with EDSS steps 5 to 10 (p=0.014) and non-RRMS (p=0.023).

**Conclusion:**

There was no association between* HLA-DR2* and MS susceptibility. However, the higher HLA-DRB5*01^+^-DRB1*1501^+^-DQB1*0602^-^ frequency may play a role in MS development. Also, HLA-DR2 did not increase significantly concerning clinical course, ethnicity, sex, and EDSS. This study further supports the importance of replication studies as susceptible loci that might differ in various ethnicities. Therefore, it is concluded that the association between *HLA-DR2* and MS is more allelic than haplotypic in Khuzestan.

## Introduction

Autoimmune diseases refer to a group of complex disorders caused by the loss of immunologic tolerance to self-antigens that lead to the immune-mediated destruction of organs and tissues. Autoimmune diseases affect up to about 5% of the world population. These diseases constitute several complex disorders, such as multiple sclerosis (MS), characterized by demyelination with axonal and neuronal degeneration (1, 2). The incidence of MS is increasing. It affects women more frequently than men and most often starts between 20 and 40 years of age (3). MS manifestations include various neurological dysfunctions, like visual and sensory problems, limb weakness, or gait disturbance. Four classical subtypes of MS include relapsing-remitting MS (RRMS), secondary progressive MS (SPMS), primary progressive MS (PPMS), and primary relapsing MS (PRMS). However, in 85% of cases, there is a clinically isolated syndrome (CIS) that later converts to RRMS (4). Studies on MS pathogenesis have indicated that an autoimmune mechanism mediated by B cells and the innate immune system has essential roles (5).

Epidemiological surveys have consistently shown that the MS frequency varies significantly worldwide. The pathogenesis and etiology of this disease are still unknown, although susceptibility to the disease depends on a complex interaction between environment and genetic factors (3). Studies on twins, adoptees, and migrants suggest that familial clustering is determined mainly by genetic factors. However, MS susceptibility is thought to be conferred by both genetic and environmental factors (3).

MS risk increases from typically 1 in 1,000 in the normal population to 1 in 4 or so for identical twins where one twin has MS. Much of this heritability is now explained and is due almost entirely to genes affecting the immune response. Over 200 genes have now been identified and, of these, 13 MHC loci and 110 non-MHC genetic loci have been detailed (6).

The human leucocyte antigen gene (*HLA*) is subdivided into three classes: I, II, and III. Classical alpha and beta chain genes, including *DP*, *DQ*, and *DR*, are located in the class II region (7). Associations of MS with alleles in *HLAs* have been identified for several decades. However, it is not yet fully understood how exactly they alter the risk of developing MS and the pathogenic mechanism (8). Associations of DRB1*1501 with MS have been consistently proven in most populations tested, although the allele does not fully explain the MS association. More studies based on genome-wide association studies have been performed for autoimmune diseases such as MS and have recognized specific *HLA-DR/DQ* genes. However, the remarkably intense linkage disequilibrium (LD) across the *HLA* region has prevented the unequivocal ascertainment of the primary disease-risk *HLA* gene. The class II association has been mapped to the DRB5*0101- DRB1*1501-DQA1*0102- DQB1*0602 haplotype in the North European population (9). These variants are almost always present together in the Caucasian population, and it is impossible to distinguish the primary association. It has been found that the robust LD mechanism in *HLA* genes is consistent with a functional epistatic interaction between DRB5*0101 and DRB1*1501 alleles. Moreover, functional epistasis is associated with a milder form of experimental autoimmune encephalomyelitis in mice ^10^. It appears that HLA-DRB1*1501, HLA-DQB1*0602, and HLA-DQA1*0102 are considered as the most critical factors, particularly in MS susceptibility (11).

The crystal structure of *HLA-DR2* complexed with a peptide from a human myelin basic protein shows that HLA proteins have various structural properties for presenting antigens based on their amino acid sequence. The *HLA* association with MS has supported the notion that MS pathogenesis is due to an autoimmune reaction, which may be against myelin-related antigens in the restricting context of DRB1*1501 (12).

The expanded disability status scale (EDSS) quantifies the MS disability level and monitors changes in the disability over time. John Kurtzke developed the scale in 1983. EDSS steps 1.0 to 4.5 refer to patients who can walk without any aid. EDSS steps 5.0 to 9.5 explain walking impairment, and death due to MS is defined by the EDSS step 10 (13). 

Pharmacogenetics refers to the study of inherited genetic variants, which can affect individual responses to drugs in terms of therapeutic and adverse effects. *HLA* is the most polymorphic gene in humans, and the heterogeneity carriage of *HLA-DR2* in MS patients reinforces the fact that MS pharmacotherapy can be targeted to speciﬁc steps in the pathogenesis pathway. Hence, identifying genetic varieties in MS patients can be of great importance. Moreover, the study of MS genetic susceptibility using the HLA ethnicity association calls attention to potential pharmacotherapy implications presented by mixed populations. 

Geographic regions and ethnicities may affect MS immunogenetics. Due to diverse ethnicities in Khuzestan in the southwest of Iran, where Iranian Arabs are mostly located, and since there are a few studies on this issue in Arabic countries, this study can be of great importance.

Here, we integrated genotypes of *HLA* alleles for the *DRB5*, *DRB1*, and *DQB1* genes concerning MS-associated variants to determine the role of putative haplotypes that alter MS susceptibility. We also analyzed the association of the *HLA-DR2 *haplotype with gender, MS type, ethnicity, and the EDSS of MS patients in Khuzestan Province.

## Materials & Methods

A cohort comprising 198 patients and 201 healthy controls, matched by geographic region, age, and gender, was used in the study. The population was composed of a case-control set of Arab and non-Arab populations in Khuzestan Province, Southwest Iran.

**MS Patients**

A total of 198 MS patients, including 154 women, 41 men, and three people with unknown gender, were diagnosed according to the revised McDonald criteria (14). Women were more likely than men to develop MS. Written informed consent was obtained from each patient before enrolling in the study. Detailed information, such as age, gender, ethnicity, positive family history, and clinical features, were provided from the Khuzestan MS Society patients, Ahvaz, Iran. A neurologist carried out clinical parameters estimation and EDSS scoring. The MS patients were divided into two groups regarding EDSS, including a scale of 0 (no disability) to 5 (more severe disability) and 5 to 10 (death due to MS). Moreover, the patients were stratified into RRMS as the most common disease course and non-RRMS groups.

**Control Group**

The study included 201 healthy individuals, of whom 125 were women, 41 were men, and 35 had unknown gender. The participants were selected from the Shafa Hospital (Ahvaz, Iran). The control participants were also informed about the study and completed a questionnaire on ethnicity, sex, age, and positive family history of autoimmune disease. The healthy individuals had no family history of autoimmune disease.

**Genotyping**

Whole blood was collected in ethylene diamine tetraacetic acid (EDTA) tubes. The extracted DNA was isolated using the standard salting-out protocol. The quality and quantity of genomic DNA were determined by agarose gel electrophoresis and NanoDrop; thus, several random genome DNAs were selected.

DNA was stored at -20°C until analysis. HLA typing was performed using the polymerase chain reaction amplification with sequence-specific primers (PCR-SSP) technique and repeated if discordant data were achieved. A set of genotypes was randomly analyzed using direct DNA sequencing.

The IMGT/HLA database (http://www.ebi.ac.uk/) was used for primer design. The primers were aligned in NCBI/blast (www.ncbi.nlm.nih.gov). The HLA-DQB1*0602, -DRBI*1501, -DRB5*01 primers used for genotyping were published previously (15-17).

Since specific PCR-SSP products assign alleles, the polymerase chain reaction (PCR) product size can help interpret the data.

Every PCR reaction is assumed to work if the internal control is amplified. The myelin oligodendrocyte glycoprotein (*MOG*) gene was used for this goal. Thus, in ideal PCR conditions, a 356 bp band must be observed. The *MOG* primers were designed by the web primer design program, batchprimer3, accessible at (http://probes.pw.usda.gov/batchprimer3/). They were also aligned in NCBI/blast. The *MOG* primers were published elsewhere (15-17).

PCR was conducted in a total volume of 25 μl containing 100-200 ng of genomic DNA, 12.5 μl master mix, 1 μl of each primer (10 μM), 2 μl dNTP (5 mM), and 8.5 μl water.

Agarose gel electrophoresis was used to determine the amplified products. Thus, 3 to 5 μl of PCR products were run on 1.5% agarose gel to observe the target band. All the analyses were performed in the Genetic Laboratory of the Shahid Chamran University of Ahwaz (Ahvaz, Iran).

**Statistical Analysis**

The frequency of HLA-DRB1*1501, -DRB5*01, and -DQB1*0602 alleles were published previously ^15-17^. The allele frequency of three allelic *DR2* haplotypes, including DRB5*01^-^-DRB1*1501^+^- DQB1*0602^+^, DRB5*01^+^ -DRB1*1501^+ ^-DQB1*0602^-^*,* and DRB5*01^+^-DRB1*1501^+^-DQB1*0602^+^*,* as well as *DR2* haplotypes, including DRB5*01^+^-DQB1*0602^-^, DRB5*01^-^-DQB1*0602^-^, DRB5*01^+^-DQB1*0602^+^, and DRB5*01^-^-DQB1*0602^+^*, *were determined as a percentage. Fisher's exact and Chi-square tests were used to compare the frequency of the mentioned genotypes between the patients and controls. SPSS statistical software (version 16, SPSS, SPSS Inc., USA) was used for data analysis. A p-value < 0.05 was considered significant.

## Results

As mentioned above, the *HLA-DR2* haplotype was evaluated among the 198 MS patients and 201 healthy age- and sex-matched individuals.

The summarized characteristics of the MS patients were published previously (15-17). 

The association and frequency of the *DR2* haplotypes, including DRB5*01^-^-DRB1*1501^+^-DQB1*0602^+^, DRB5*01^+^-DRB1*1501^+^-DQB1*0602^-^, DRB5*01^+^-DRB1*1501^+^-DQB1*0602^+^, DRB5*01^+^-DQB1*0602^-^, DRB5*01^-^-DQB1*0602^-^, DRB5*01^+^-DQB1*0602^+^, and DRB5*01^-^-DQB1*0602^+^, were investigated in the MS patients and compared with the healthy controls (Table 1). DRB5*01^-^-DQB1*0602^-^ was the most frequent variant between the patients and controls. However, no significant difference was found concerning the mentioned haplotypes between the two groups (39.26% vs. 36.31% p = 0.547). It should be noted that the frequency of DRB5*01+-DRB1*1501+-DQB1*0602- was also low in both patients and controls (0%).

Moreover, no significant correlation was observed among the mentioned variants with Fars and Arab ethnics (Table 2 and 3). It was found that no one carried DRB5*01^+^-DRB1*1501^+^-DQB1*0602^-^ and DRB5*01^+^-DQB1*0602^-^ haplotypes in controls (0%).

Furthermore, the HLA-DRB5*01^+^-DRB1*1501^+^-DQB1*0602^-^ haplotype was positively associated with EDSS steps 5 to 10 (12.5% vs. 1.15%, p= 0.014), although no more association was found between the rest of the haplotypes and level of disability, as shown in Table 4.

As shown in Table 5, the mentioned variants demonstrated no significant correlation with RRMS as the most common disease course except for DRB5*01^+^-DRB1*1501^+^-DQB1*0602^-^ that showed a positive association with non-RRMS (11.11% vs. 1.16%, p = 0.023).

We also studied the association of *HLA-DR2* haplotypes with MS in the females and males separately. There was no association between genotype frequencies of these haplotypes with gender. It was found that no male MS patient carried the DRB5*01^+^-DRB1*1501^+^-DQB1*0602^-^ and DRB5*01^+^-DQB1*0602^-^ haplotypes.

## Discussion

MS is a complex neurodegenerative and multifactorial disease. The exact cause of the disease is still unclear, but it probably results from the interaction of genetic, epigenetic, and environmental factors and intrinsic factors, and epistatic effects (e.g., gene-gene interactions). The findings implicate that DRB5*01^-^-DQB1*0602^-^ usually has a high frequency in the studied population, while DRB5*01^+^-DRB1*1501^+^-DQB1*0602^-^ and DRB5*01^+^-DQB1*0602^-^ are rare in Khuzestan, in both sex and ethnic groups. Thus, frequency differences between the studied groups are not meaningful. 

The present study aimed to evaluate the frequency of and the association between DRB5*01^-^-DRB1*1501^+^-DQB1*0602^+^, DRB5*01^+^-DRB1*1501^+^-DQB1*0602^-^, DRB5*01^+^-DRB1*1501^+^-DQB1*0602^+^, DRB5*01^+^-DQB1*0602^-^, DRB5*01^-^-DQB1*0602^-^, DRB5*01^+^-DQB1*0602^+^, and DRB5*01^-^-DQB1*0602^+^ haplotypes and to assess the effect of the *HLA-DR2* haplotype on MS susceptibility in Khuzestan Province. Since no association was found between the *HLA-DR2* variants and MS susceptibility, most of the mentioned haplotypes had low frequency in Southwest Iran. It was concluded that these haplotypes might not affect the MS development. However, MS is a complex, heterogenic, multifactorial disease with an unknown etiology. Thus, other factors like recombination, genetic drift, latitude, epistasis, and founder effect might affect the MS pathogenesis.

Subsequently, numerous studies have been conducted on the association between HLA antigens and MS. In several studies, the role of the *HLA* class II, especially DQA1*0102, DQB1*0602, DRB1*1501, and DRB5*01, has been hypothesized to be the primary HLA genetic susceptibility factor for MS susceptibility ^4^^,^^17^. A study on many Swedish MS patients showed that the *HLA-DR2* haplotype was overrepresented among the patients (18).

On the other hand, association studies in some populations have confirmed that the DRB1*1501 allele itself determines MS-associated susceptibility (19). While in other populations, there are various risk variants, or they do not contain only the DRB1*1501 allele; as in the Sardinia population where MS was correlated with the HLA-DRB1*0405-DQA1*0501-DQB1*0301 and HLA-DRB1*0301-DQA1*0501-DQB1*0201 haplotypes (20). A partial explanation for strong linkage disequilibrium in the *HLA *was provided by epistasis among the *HLA* class II (*DRB1*, *DQA1*, and *DQB1*) alleles in human immune responses. It has relevance to animal models, as well. Also, *DRB1*, *DQA1*, and *DQB1* alleles contribute to MS susceptibility, although epistatic interactions suggest haplotypic rather than allelic HLA association (21). 

The association of MS with the HLA-DRB1*1501-DQB1*0602 haplotype was repeatedly demonstrated in high-risk northern European populations. It is unclear whether the effect is explained by the *HLA-DQB1* or *-DRB1* gene within the susceptibility haplotype, which are in strong LD. African American family-based and case-control association studies on MS revealed that a primary role for the *DRB1* locus was independent of DQB1*0602. Thus, it was unlikely to be solely explained by admixture since a substantial proportion of the susceptibility chromosomes from African American patients with MS displayed haplotypes consistent with an African origin (22).

HLA-DQB1*0602 determined MS susceptibility in a humanized MS model in HLA-DRB1*1501; -DQB1*0602 transgenic mice. A recent work in humanized mice indicated functional epistatic interactions whereby DRB5*0101 directly modulates the severity of the ensuing disease through the activation-induced cell death of encephalitogenic T cells, which are restricted by DRB1*1501. The *HLA-DR15* haplotype (DRB1*1501, DRB5*0101, and DQB1*0602) has been identified as the most substantial susceptibility factor for MS (23, 24). On the contrary, no correlation was found in the current study between HLA-DRB1*1501, -DRB5*0101, and -DQB1*0602 with MS.

A study on Afro-Brazilians showed that DQB1*0602 was associated with the disease in the absence of the DRB1*1501 allele (25). However, no association was observed between MS with carrying DRB1*1501 and DQB1*0602 in our survey. As in studies on the current population, the positive association of DRB1*1501 allele and the lack of DQB1*0602 association with MS were found in Khuzestan Province (15, 16). Thus, it is likely that the association with the disease is more allelic than haplotypic in these ethnic groups. 

Contrary to this study, an HLA-DR2-related haplotype implicated that the HLA-DRB1*1501-DRB5*01-DQB1*0602-DQA1*0102 haplotype was susceptible to MS (26).

This study aimed to examine part of MS genetic background in Khuzestan Province. In the present study, no association was observed between the MS patients and healthy controls regarding the HLA-DR2-related haplotype. Thus, it is thought that MS is more allelic than haplotypic. It should be noted that as MS is a polygenic disease, some other haplotypes in other genes probably are associated with MS susceptibility. The differences observed between our results and other populations could be due to discrepancies in the genetic pool of the populations, latitude, and environmental factors. A small sample size may affect the statistical power. Therefore, our data need confirmation in larger sample size assays.

On the other hand, the population of Khuzestan Province is heterogeneous and consists of various ethnicities like Lor, Arab, Kord, and Fars. Additional polymorphisms exist in the *HLA* gene that might contribute to the susceptibility or activity of MS in this population. A larger number of patients and control subjects are required to verify the results. In conclusion, this study supports the importance of replication studies as susceptible loci that might differ in various ethnic groups. It is suggested to type more haplotypes in other MS populations, especially in regions considered high risk for MS, to achieve more documented data.

**Table 1:** Association of DR2 haplotype with MS in Khuzestan province

	HLA haplotypes	Patients		Controls		p value
		posetive haplotype (%)	Total	posetive haplotype (%)	Total	
1	DRB5*01^+^-DQB1*0602^-^	6 (3.27)	183	4 (2.08)	192	0.452
2	DRB5*01^-^-DQB1*0602^-^	75 (39.26)	191	73 (36.31)	201	0.547
3	DRB5*01^+^-DQB1*0602^+^	44 (23.78)	185	34 (18.27)	186	0.184
4	DRB5*01^-^-DQB1*0602^+^	62 (33.87)	183	68 (35.23)	193	0.738
5	DRB5*01^+^-DRB1*1501^+^-DQB1*0602^+^	21 (10.79)	195	17 (10.30)	165	0.886
6	DRB5*01^+^ -DRB1*1501^+ ^-DQB1*0602^-^	3 (1.51)	198	0 (0)	174	0.103
7	DRB5*01^-^-DRB1*1501^+^- DQB1*0602^+^	26 (13.54)	192	14 (8.18)	171	0.104

*=significant P values; Statistical significance was at p < 0.05. Values are expressed as No. (%).

**Table 2:** Association of DR2 haplotype with Fars ethnic in MS patients of Khuzestan province

	HLA haplotypes	Patients		Controls		p value
		posetive haplotype (%)	Total	posetive haplotype (%)	Total	
1	DRB5*01^+^-DQB1*0602^-^	6 (5.08)	115	2 (2.53)	79	0.355
2	DRB5*01^-^-DQB1*0602^-^	39 (33.91)	115	26 (32.09)	81	0.790
3	DRB5*01^+^-DQB1*0602^+^	29 (25)	116	11 (15.71)	70	0.135
4	DRB5*01^-^-DQB1*0602^+^	40 (34.78)	115	33 (42.85)	77	0.259
5	DRB5*01^+^-DRB1*1501^+^-DQB1*0602^+^	12 (10.71)	112	6 (7.69)	78	0.484
6	DRB5*01^+^ -DRB1*1501^+ ^-DQB1*0602^-^	2 (1.62)	123	0 (0)	79	0.255
7	DRB5*01^-^-DRB1*1501^+^- DQB1*0602^+^	16 (13.11)	122	5 (7.46)	67	0.273

*=significant P values; Statistical significance was at p < 0.05. Values are expressed as No. (%).

**Table 3:** Association of DR2 haplotype with Arab ethnic in MS patients of Khuzestan province

	HLA haplotypes	Patients		Controls		p value
		posetive haplotype (%)	Total	posetive haplotype (%)	Total	
1	DRB5*01^+^-DQB1*0602^-^	1 (1.40)	71	0 (0)	88	0.267
2	DRB5*01^-^-DQB1*0602^-^	32 (44.44)	72	34 (40.96)	83	0.662
3	DRB5*01^+^-DQB1*0602^+^	14 (20.58)	68	15 (19.73)	76	0.899
4	DRB5*01^-^-DQB1*0602^+^	23 (33.33)	69	28 (35)	80	0.831
5	DRB5*01^+^-DRB1*1501^+^-DQB1*0602^+^	7 (9.72)	72	9 (10.84)	83	0.819
6	DRB5*01^+^ -DRB1*1501^+ ^-DQB1*0602^-^	1 (1.25)	80	0 (0)	89	0.290
7	DRB5*01^-^-DRB1*1501^+^- DQB1*0602^+^	9 (12)	75	8 (22.85)	35	0.142

**Table 4:** Association of HLA DR2 with EDSS in MS patients of Khuzestan province

	HLA haplotypes	EDSS 1-5		EDSS 5-10		p value
		posetive haplotype (%)	Total	posetive haplotype (%)	Total	
1	DRB5*01^+^-DQB1*0602^-^	5 (3.10)	161	1 (14.28)	7	0.119
2	DRB5*01^-^-DQB1*0602^-^	55 (34.59)	159	5 (62.05)	8	0.108
3	DRB5*01^+^-DQB1*0602^+^	41 (25.78)	159	0 (0)	8	0.098
4	DRB5*01^-^-DQB1*0602^+^	55 (34.37)	160	2 (25)	8	0.585
5	DRB5*01^+^-DRB1*1501^+^-DQB1*0602^+^	19 (10.79)	176	0 ()	8	0.326
6	DRB5*01^+^ -DRB1*1501^+ ^-DQB1*0602^-^	2 (1.15)	173	1 (12.5)	8	0.014^*^
7	DRB5*01^-^-DRB1*1501^+^- DQB1*0602^+^	25 (15.06)	166	2 (25)	8	0.448

*=significant P values; Statistical significance was at p < 0.05. Values are expressed as No. (%).

**Table 5:** Analysis of association between HLA haplotypes and RRMS

	HLA haplotypes	RRMS		Non-RRMS		p value
		posetive haplotype (%)	Total	posetive haplotype(%)	Total	
1	DRB5*01^+^-DQB1*0602^-^	5 (3.10)	161	1 (11.11)	9	0.205
2	DRB5*01^-^-DQB1*0602^-^	65 (3.86)	168	3 (33.33)	9	0.748
3	DRB5*01^+^-DQB1*0602^+^	40 (24.53)	163	1 (11.11)	9	0.357
4	DRB5*01^-^-DQB1*0602^+^	56 (34.78)	161	4 (44.44)	9	0.555
5	DRB5*01^+^-DRB1*1501^+^-DQB1*0602^+^	20 (11.56)	173	0 (0)	9	0.280
6	DRB5*01^+^ -DRB1*1501^+ ^-DQB1*0602^-^	2 (1.16)	172	1 (11.11)	9	0.023^*^
7	DRB5*01^-^-DRB1*1501^+^- DQB1*0602^+^	25 (14.88)	168	2 (22.22)	9	0.551

*=significant P values; Statistical significance was at p < 0.05. Values are expressed as No. (%).

**Table 6:** Association of HLA haplotype with female in MS patients of Khuzestan province

	HLA haplotypes	Patients		Controls		p value
		posetive haplotype (%)	Total	posetive haplotype (%)	Total	
1	DRB5*01^+^-DQB1*0602^-^	6 (4.20)	149	2 (20.06)	97	0.396
2	DRB5*01^-^-DQB1*0602^-^	53 (39.84)	133	36(33.96)	106	0.350
3	DRB5*01^+^-DQB1*0602^+^	35 (23.80)	147	18 (14.47)	103	0.228
4	DRB5*01^-^-DQB1*0602^+^	48 (32.65)	147	43 (40.95)	105	0.176
5	DRB5*01^+^-DRB1*1501^+^-DQB1*0602^+^	14 (9.79)	143	13 (10.40)	125	0.869
6	DRB5*01^+^ -DRB1*1501^+ ^-DQB1*0602^-^	3 (1.94)	154	0 (0)	107	0.146
7	DRB5*01^-^-DRB1*1501^+^- DQB1*0602^+^	18 (12.16)	148	7 (7.52)	93	0.251

*=significant P values; Statistical significance was at p < 0.05. Values are expressed as No. (%).

**Table 7:** Association of HLA haplotype with male in MS patients of Khuzestan province

	HLA haplotypes	Patients		controls		p value
		posetive haplotype (%)	Total	posetive haplotype (%)	Total	
1	DRB5*01^+^-DQB1*0602^-^	0 (0)	40	1 (3.03)	33	0.268
2	DRB5*01^-^-DQB1*0602^-^	17 (41.46)	41	15 (36.58)	41	0.651
3	DRB5*01^+^-DQB1*0602^+^	8 (21.05)	38	6 (17.14)	35	0.672
4	DRB5*01^-^-DQB1*0602^+^	14 (35.89)	39	12 (34.28)	35	0.885
5	DRB5*01^+^-DRB1*1501^+^-DQB1*0602^+^	4 (10.52)	38	1 (2.50)	40	0.077
6	DRB5*01^+^ -DRB1*1501^+ ^-DQB1*0602^-^	0 (0)	39	0 (0)	40	-
7	DRB5*01^-^-DRB1*1501^+^- DQB1*0602^+^	8 (20)	40	5 (15.62)	32	0.632

*=significant P values; Statistical significance was at p < 0.05. Values are expressed as No. (%).
